# Temporal and situational analysis of 7-m shots in elite handball: a multi-season study

**DOI:** 10.3389/fpsyg.2026.1811808

**Published:** 2026-04-13

**Authors:** Hikmet Gümüş, Celal Gençoglu, Yücel Makaracı, Zarife Pancar, Ilknur Yazıcılar Özçelik

**Affiliations:** 1 Department of Coaching Education, Faculty of Sport Sciences, Dokuz Eylul Universitesi, Alsancak, Türkiye; 2 Department of Physical Education and Sports, Faculty of Sport Sciences, Dokuz Eylül University, Alsancak, Türkiye; 3 Department of Coaching Education, Faculty of Sports Sciences, Karamanoglu Mehmetbey Universitesi, Karaman, Türkiye; 4 Department of Physical Education and Sports Teaching, Faculty of Sports Sciences, University of Gaziantep, Gaziantep, Türkiye; 5 Department of Physical Education and Sports, Faculty of Education, Amasya Universitesi, Amasya, Türkiye

**Keywords:** 7-m penalty, close matches, goalkeeper performance, handball, throwing efficiency

## Abstract

The 7-m shot is a critical scoring opportunity in elite handball, significantly impacting match outcomes. Despite existing research on performance metrics and tactical elements, a gap remains in understanding how game timing and situational context influence 7-m shot outcomes. This study addresses this by analyzing 7-m shots across different game phases, under pressure conditions, and considering temporal and contextual factors. Utilizing play-by-play data from 2,978 matches in the Danish Handball top-tier Men's and Women's leagues (2017–2024 seasons), the study examined 38,480 penalty-related events. Temporal analysis allocated “penalty awarded” events to 5-min intervals, and success rates were calculated for various scenarios. Close matches were defined by a ≤ 2-goal difference at regulation time, and “clutch moments” were the final 5 min of play. Results indicate a consistent temporal pattern, with penalty occurrences typically peaking in the central phases of each half. Overall success rates for 7-m shots remained stable across seasons and genders, ranging from 73.4% to 78.1% in women's and 75.6% to 77.7% in men's competitions. No statistically significant difference was found in success rates between men's and women's leagues. Performance in the final 5 min showed divergent gender patterns: women equalled or exceeded their whole-match baseline in five of seven seasons (average: +2.7 pp), while men more frequently recorded negative differences (average: −1.4 pp), with the notable exception of 2023–24 (+6.9 pp). Goalkeeper saves accounted for 74.6% of all unsuccessful penalties across both groups, with higher rates in men's leagues (78.9%) than women's leagues (70.9%). These findings suggest that elite handball players can maintain stable execution of 7-m shots despite situational pressure, supporting the role of effective psychological self-regulation in high-stakes performance. Integrating psychological skills training, such as attentional focus strategies and stress regulation, alongside technical preparation may enhance penalty-shot training and match preparation.

## Introduction

In elite handball, the 7-m penalty shot represents a critical scoring opportunity that can decisively influence match outcomes. Awarded following clear defensive infractions, these penalty shots provide a unique performance context: relatively controlled conditions with minimal defensive interference, yet executed under heightened psychological pressure (Debanne et al., 2018). Research consistently demonstrates that 7-m shots are among the most effective scoring methods in handball, with winning teams exhibiting significantly higher conversion rates than losing teams (Srhoj et al., 2001; Vuleta et al., 2003). In a regression analysis of 12 performance indicators from the 2000 Men's European Championship, Vuleta et al. (2003) identified 7-m throw efficiency as a key differentiator between winning and losing teams. Similarly, Oliveira et al. (2012) found that 7-m efficiency distinguished between match outcomes in the Spanish professional league, even after controlling for other high-percentage shot types, such as 6-m and 9-m attempts. These findings underscore the strategic importance of penalty shot performance in competitive handball.

Despite accounting for only 6%−9% of total shots in handball matches (Wagner et al., 2011), 7-m penalties exert disproportionate influence on match outcomes due to their high conversion rates and occurrence during critical match phases. Success rates for 7-m penalties typically range between 68% and 84% across competitive levels (Meletakos et al., 2024), with elite competitions consistently reporting conversion rates above 70% (Meletakos et al., 2011). For instance, Meletakos et al. (2011) documented stable success rates across major championships: 72.0% in 2005, 73.0% in 2007, and 71.2% in 2009. Elite goalkeepers typically maintain save rates of approximately 30% during penalty situations (Rojas et al., 2012), reflecting the inherent advantage afforded to shooters due to limited response time and optimal shooting distance. This creates a performance paradox: while 7-m shots represent high-probability scoring opportunities under controlled conditions, they occur in moments of elevated competitive pressure—particularly during close matches or final minutes of play—where even small decrements in execution can prove decisive.

The successful execution of 7-m penalty shots under competitive pressure depends not only on technical proficiency but also on athletes' capacity to regulate psychological and physiological states in high-stakes situations. Penalty contexts are characterized by temporal constraint, outcome salience, and heightened cognitive and emotional demands (Beilock and Carr, 2005; Laxdal et al., 2024). From a self-regulation perspective, players must effectively manage attentional focus, arousal levels, emotional responses, and motor execution within a compressed time window, often while experiencing intense situational pressure. The ability to maintain stable performance under such conditions has been linked to psychological resilience, neural efficiency, and adaptive psychophysiological regulation—central themes in contemporary sport psychology research (Gross, 2015; Makaraci et al., 2024). Psychophysiological research has demonstrated that skilled performers allocate neural resources more efficiently during high-demand motor tasks, as reflected in lower cortical activation per unit of performance output. Superior motor performance is characterized by the selective functional activation of task-relevant neural processes and the suppression of task-irrelevant neuromotor noise during motor preparation. Specifically, Li et al. (2025) demonstrated that successful football penalty kicks under difficult task conditions were associated with lower alpha power in frontal and central regions during motor preparation, reflecting efficient neural resource allocation for motor planning and control, consistent with the psychomotor efficiency hypothesis. Similarly, Lu et al. (2025) compared amateur and novice golfers during a visuomotor task and found that amateurs exhibited higher alpha power in frontal, parietal, and temporal regions than novices, indicating reduced motor programming effort, less verbal-analytical engagement, and more refined attentional control. Together, these findings highlight the role of neural efficiency and brain–behavior mechanisms in sustaining motor performance under demanding conditions.

Empirical evidence on how elite players regulate performance under pressure remains mixed. Alsharji (2014) and Debanne et al. (2018) have emphasized the impact of psychological pressure and cognitive style on penalty shot efficacy, suggesting that stress may impair execution. Conversely, Bühren and Gabriel (2023) analyzed over 5,500 penalty throws from a single season in German handball and found that performance often improved during decisive moments, suggesting that some players effectively channel pressure to their advantage. These conflicting findings highlight the need for further investigation into how situational and temporal factors interact with self-regulation processes during penalty execution.

Despite extensive research on handball performance, 7-m shots are often treated as homogeneous events, with limited consideration of how temporal context and competitive pressure influence execution stability. Previous studies have identified multiple determinants of penalty shot performance, including anticipatory demands and temporal limitations faced by goalkeepers (Huesmann et al., 2023), biomechanical precision in shot execution (Van den Tillaar and Ettema, 2009), ball velocity and accuracy (Demiray et al., 2025; García et al., 2017), shot type selection and target location, and contextual factors such as home vs. away conditions (Hantǎu and Hantǎu, 2014). However, few studies have systematically examined whether performance stability during 7-m shots reflects effective self-regulation under pressure or whether execution deteriorates during high-stakes moments such as close-score scenarios or final minutes of play. This gap limits understanding of how elite players adapt to cognitive, emotional, and physiological demands in critical performance contexts.

Accordingly, the present study provides a comprehensive situational and temporal analysis of 7-m penalty shots in elite handball across multiple seasons. Specifically, it examines: (a) the temporal distribution of penalty shots across match phases, (b) success rates under pressure-laden conditions such as close matches and clutch moments (final 5 min of regulation time), and (c) the contribution of goalkeeper intervention to unsuccessful outcomes. We hypothesize that (a) the frequency of 7-m penalty shots could vary significantly across match phases, (b) penalty shot success rates could be significantly lower during the last 5 min of the match, and (c) goalkeeper contribution could be a significant factor in penalty failure. By integrating situational performance indicators with a pressure-sensitive framework, this study contributes to a more ecologically valid understanding of performance regulation in elite sport and offers applied insights relevant to psychological preparation, decision-making, and performance optimization under competitive stress.

## Methods

### Dataset description

This study employed publicly accessible match analysis data obtained from https://tophaandbold.dk/, the platform officially endorsed by the Danish Handball Federation. The dataset comprises play-by-play match analysis from the top-tier Danish professional men's and Women's handball leagues. It includes detailed event-level records from official league matches played between the 2017 and 2024 seasons. For each match, event entries include the exact time (in seconds) from the beginning of the game, the type of event (e.g., goal, turnover, penalty awarded), and team-specific identifiers. Each event is timestamped and categorized, enabling temporal mapping of in-game actions, including shot attempts, fouls, timeouts, and penalties. The dataset is organized by season and segregated by gender, covering both the men's and women's divisions. This temporal resolution allows for detailed within-match analyses and facilitates comparisons across different phases of play. The dataset comprises 2,978 unique matches, providing a robust sample for statistical analysis (Table 1). Penalty-related events are a key focus, with 38,480 such events identified. These include all events where the event description contains the word “penalty” (e.g., penalty awarded, penalty goal, penalty saved, etc.). This comprehensive dataset allows for in-depth quantitative and comparative analysis of penalty performance, goalkeeper effectiveness, and other match dynamics across genders and seasons.

**Table 1:** Included match numbers according to gender and season.

Season	The men's league	The women's league	Total
17–18 season	222	168	390
18–19 season	227	226	453
19–20 season	168	165	333
20–21 season	198	228	426
21–22 season	258	226	484
22–23 season	231	201	432
23–24 season	231	229	460

### Data selection and filtering

The present analysis focused specifically on penalty-related events classified as “Penalty awarded”, “Penalty goal”, “Penalty saved”, “Penalty on-post”, “Penalty miss off-target”, referring to the awarding of 7-m and outcomes of the shots.

Temporal Analysis Procedures: To examine the distribution of penalty awards over the course of a match, each “*penalty awarded*” event was allocated to a 5-min match interval (e.g., 0–4:59, 5–9:59, ..., 55–59:59). This binning process was applied uniformly across all matches within the selected season and gender subset. Aggregated counts of penalty events per interval were computed, and the results were expressed as both absolute frequencies and percentages of the total number of awarded penalties. This temporal stratification facilitated the identification of patterns, such as the clustering of penalties in specific match segments (e.g., end-of-half periods).

Penalty Shot Success and Failure Rate Analysis: The effectiveness of penalty shot analysis was evaluated using the event type “Penalty awarded” as the indicator for initiating a 7-m shot opportunity. Each occurrence of “Penalty awarded” was treated as a single penalty shot attempt, irrespective of the eventual outcome. The outcome of each penalty attempt was categorized as either successful or unsuccessful, based on the subsequent event recorded. A “Penalty goal” was defined as a successful execution of the penalty shot. In contrast, the following event types were collectively treated as unsuccessful outcomes: “Penalty saved”, “Penalty on post”, and “Penalty missed – off target”. The success rate of penalty shots was calculated as the ratio of successful penalty goals to the total number of penalty attempts awarded. Specifically, it was computed using the formula:


$$\begin{array}{l} Success\;rate=Number\;of\;penalty\;goals/total\;number\;of\\ penaltyattemptsawarded\\ Failure\;Rate\;=\;1-\;Success\;Rate \end{array}$$

Penalty Shot Performance in Close Matches: To investigate the influence of match competitiveness on penalty shot outcomes, a subset analysis was conducted focusing on “close matches.” For this study, close matches were defined as those in which the absolute goal difference at the end of regulation time was two goals or fewer. This threshold was selected to represent matches where outcomes were most uncertain and in-game pressure likely elevated. Within the close match subset, the performance of penalty shots was assessed by calculating both the success rate and failure rate as described in the previous section.

Penalty Shot Performance in Clutch Moments: To assess the effect of time-dependent match pressure on penalty shot performance, a focused analysis was conducted on clutch moments, defined as the final 5 min of regulation time. The aim was to determine whether temporal proximity to the end of the match influenced the success or failure rates of penalty shots. Using timestamped play-by-play data, all “Penalty awarded” events occurring within the final 5-min interval were identified. These rates were compared against two reference distributions: all matches and within the last 10 min of play, allowing for temporal trend analysis across progressively high-pressure match phases.

Goalkeeper Involvement in Unsuccessful Penalty Shots: To further elucidate the underlying factors contributing to unsuccessful penalty attempts, an additional analysis was conducted to quantify goalkeeper involvement across seasons. Specifically, each unsuccessful penalty event was classified according to its cause: (a) goalkeeper intervention (“Penalty saved”), or (b) player-induced error (“Penalty on post” or “Penalty missed—off target”). For each season, the proportion of unsuccessful penalties resulting from goalkeeper saves was calculated relative to the total number of unsuccessful attempts. This measure, expressed as a percentage, provided an indicator of the extent to which goalkeeper performance contributed to missed penalties, thereby distinguishing between defensive success and execution errors by the shooter.

### Statistical analysis

Descriptive statistics were used to summarize the temporal distribution and success rates of 7-m penalty shots across seasons and genders. Differences in penalty success rates between men's and women's leagues across seasons were examined using the Mann–Whitney *U*-test due to the non-parametric nature of the aggregated percentage data. To assess the influence of temporal pressure on performance, success rates during clutch situations (final 5 min of play) were compared with whole-match success rates using the chi-square (χ^2^) test. All tests were two-tailed. All statistical analyses were conducted using IBM SPSS Statistics for Windows (Version 25.0; IBM Corp., Armonk, NY, USA). The level of statistical significance was set *a priori* at *p* < 0.05.

## Results

Table 2 presents the temporal distribution of 7-m penalties across 5-min intervals across all seasons and genders. Across all seasons, men's competitions recorded higher total penalty counts than women's in six of the seven seasons examined, with the sole exception being 2021–22, where both cohorts reached their respective maxima. The 2019–20 season produced the lowest total penalties in both gender groups (972 in women's and 1,022 in men's competitions), a convergent decline most plausibly attributable to pandemic-related disruptions to competition schedules and match volumes during that period. Men's competitions consistently produced higher peak counts than women's across most seasons, consistent with the higher total penalty volumes reported above. Average penalties per interval followed a broadly parallel pattern, ranging from 74.8 (Women's 2019–20) to 118.6 (Women's 2021–22) in women's competitions, and from 78.6 (Men's 2019–20) to 135.2 (Men's 2021–22) in men's competitions.

**Table 2:** Temporal analysis of 7-m penalties, both male and female leagues across seven seasons long.

Season and gender	Total penalties	Peak interval start	Peak count	Average per interval
Women's 2017–2018	1,121	55	105	80.1
Women's 2018–2019	1,467	40	143	112.8
Women's 2019–2020	972	55	94	74.8
Women's 2020–2021	1,523	25	147	117.2
Women's 2021–2022	1,542	10	152	118.6
Women's 2022–2023	1,161	25	127	89.3
Women's 2023–2024	1,384	15	134	106.5
Men's 2017–2018	1,528	40	143	117.5
Men's 2018–2019	1,550	35	157	119.2
Men's 2019–2020	1,022	5	96	78.6
Men's 2020–2021	1,466	55	137	112.8
Men's 2021–2022	1,758	40	166	135.2
Men's 2022–2023	1,342	15	132	103.2
Men's 2023–2024	1,397	45	133	107.5

Table 3 presents a comprehensive breakdown of 7-m penalty shot outcomes across seven consecutive seasons (2017–18 to 2023–24) for both women's and men's competitions, reporting awarded penalties, goals, saves, post attempts, missed shots, total unsuccessful outcomes, and overall success and failure rates. Penalty success rates demonstrated high stability across all seasons and both gender groups, ranging from 73.4% to 78.1% in women's competitions and 75.6% to 77.3% in men's competitions. Correspondingly, failure rates ranged from 21.9% to 26.6% in women's leagues and from 22.3% to 24.4% in men's leagues.

**Table 3:** Handball 7-m penalty shot success rates across seasons and genders.

Season and gender	Penalty awarded	Penalty goal	Penalty saved	Penalty post	Penalty missed	Total-unsuccessful	Success rate %	Failure rate %
Women's 2017–2018	1,121	851	193	54	23	270	75.9	24.1
Women's 2018–2019	1,467	1,113	255	69	30	354	75.9	24.1
Women's 2019–2020	972	733	171	47	21	239	75.4	24.6
Women's 2020–2021	1,523	1,118	284	87	34	405	73.4	26.6
Women's 2021–2022	1,542	1,200	251	60	31	342	77.8	22.2
Women's 2022–2023	1,161	905	171	59	26	256	78	22
Women's 2023–2024	1,384	1,081	213	62	28	303	78.1	21.9
Men's 2017–2018	1,528	1,155	295	54	24	373	75.6	24.4
Men's 2018–2019	1,550	1,183	287	47	33	367	76.3	23.7
Men's 2019–2020	1,022	774	202	29	17	248	75.7	24.3
Men's 2020–2021	1,466	1,133	271	48	14	333	77.3	22.7
Men's 2021–2022	1,758	1,338	340	60	20	420	76.1	23.9
Men's 2022–2023	1,342	1,028	253	45	16	314	76.6	23.4
Men's 2023–2024	1,397	1,085	221	56	35	312	77.7	22.3

A comparison of penalty success rates in men's and women's leagues across the evaluated seasons showed no statistically significant difference (*U* = 28.0, *p* = 0.71) according to the Mann–Whitney *U*-test. The mean success rate for 7-m penalties indicates that, despite minor differences in variability, the overall performance in terms of penalty success is remarkably similar between genders. In summary, the results suggest that penalty shot success rates are stable across seasons and exhibit no meaningful statistical differences between men's and women's leagues.

**Figure 1:**
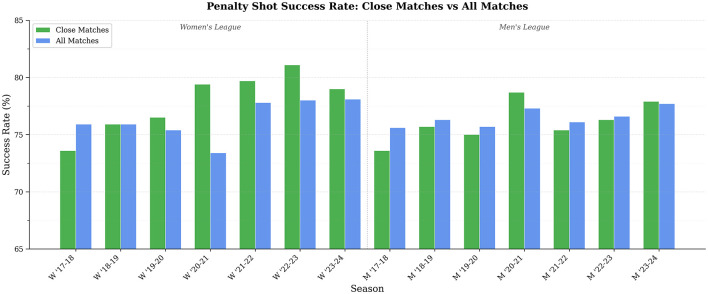
Penalty shot performance in close matches: success rate comparison.

The analysis presented in Figure 1 provides insights into the penalty success rates during close matches compared to all matches. The data reveals fluctuations across different seasons, indicating that certain periods exhibit marginally elevated or diminished success rates when penalties are taken under pressure conditions. This highlights the potential impact of situational factors on performance in high-stakes scenarios.

**Figure 2:**
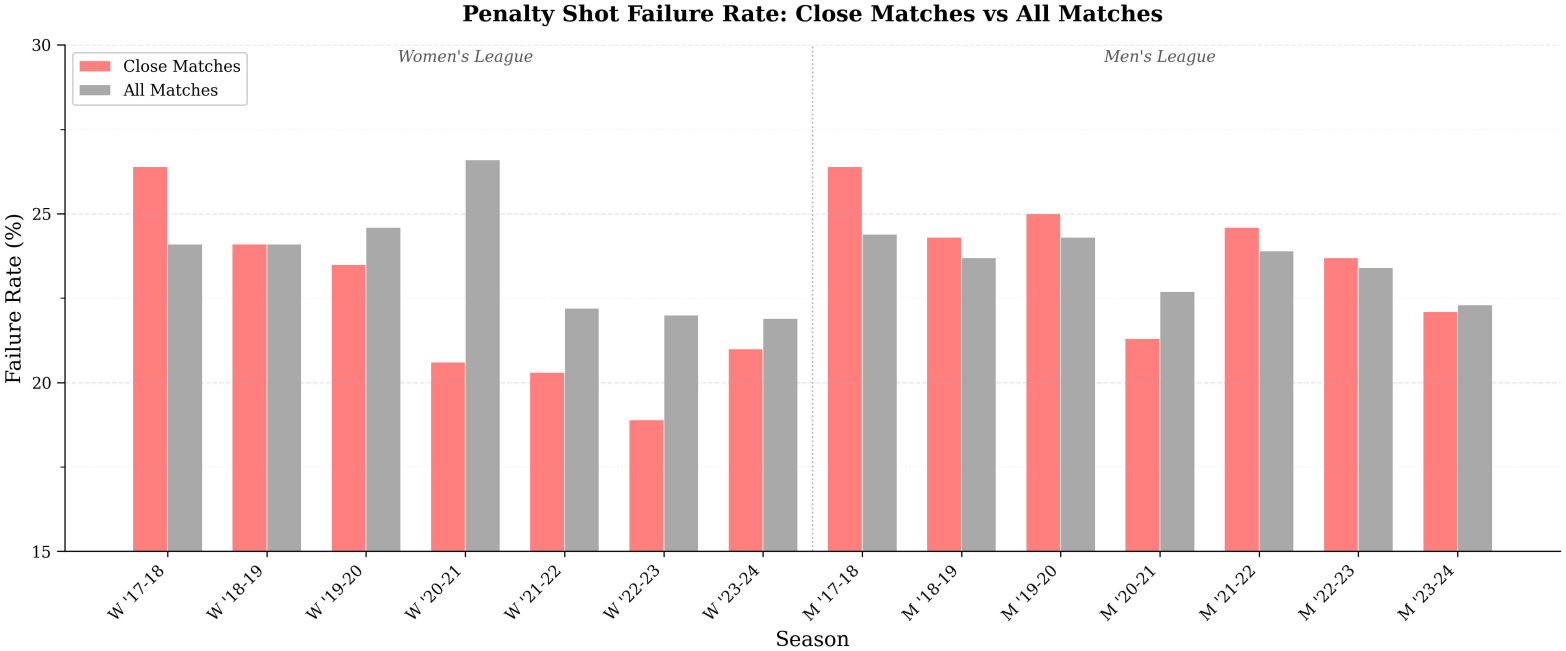
Penalty shot performance in close matches: failure rate comparison.

Figure 2 illustrates the penalty shot failure rates for close and all matches along seven consecutive seasons in elite handball leagues. These findings suggest that the success and failure patterns of penalty shots differ between closely contested games and broader matches, highlighting the contextual influence of match competitiveness on penalty performance.

Table 4 presents a summary of the number of penalties awarded along with their respective success rates for each time window. It also highlights the difference in success rates between the last 5 min of the match and the entire match. However, the chi-square test indicated no significant difference between penalty-shot success rates in the final 5 min of play and those across the entire match (χ^2^ = 5.51 × 10^−5^, *p* = 0.994).

**Table 4:** Success rate of 7-m penalty shots during the clutch moments of the game and the whole match.

Season and gender	Last5-min awarded	Last5-min success %	Last 10-min success %	All match success %	Last5-min vs. all match difference
Women's 2017–2018	107	81.3	74.3	75.6	5.7
Women's 2018–2019	139	80.6	79.6	75.7	4.9
Women's 2019–2020	98	75.5	75.8	75.4	0.1
Women's 2020–2021	143	73.4	73.8	73.3	0.1
Women's 2021–2022	139	81.3	77.2	77.8	3.5
Women's 2022–2023	95	77.9	775	78	−0.1
Women's 2023–2024	116	77.6	75.6	77.9	−0.3
Men's 2017–2018	135	71.9	76	75.8	−3.9
Men's 2018–2019	153	73.9	75.2	76.5	−2.6
Men's 2019–2020	76	77.6	75.6	76	1,6
Men's 2020–2021	140	75.7	74.7	77.3	−1.6
Men's 2021–2022	153	68.6	73.7	76.1	−7.5
Men's 2022–2023	118	73.7	72.9	76.4	−2.7
Men's 2023–2024	121	84.3	81.9	77.4	6.9

**Figure 3:**
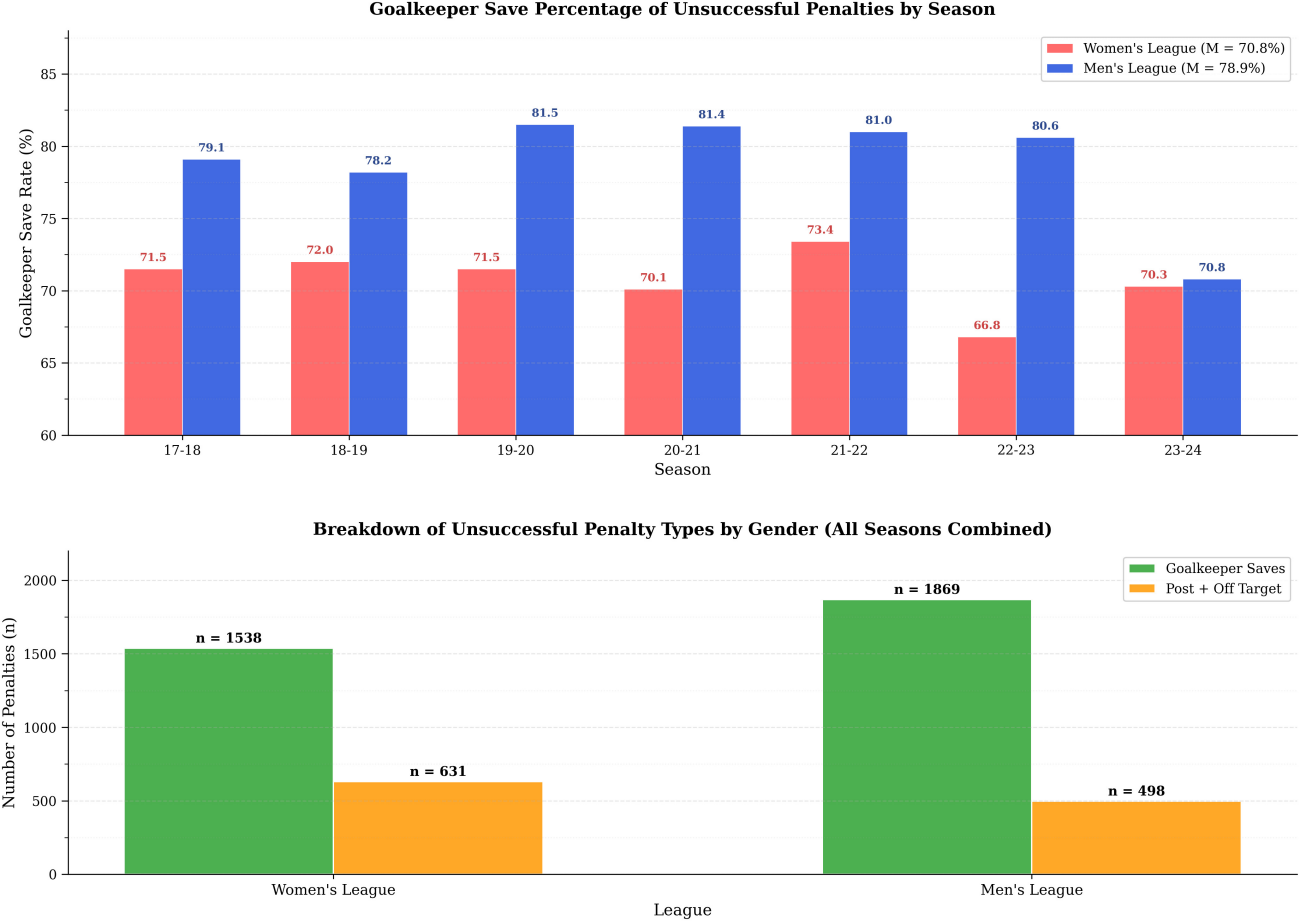
Goalkeeper involvement in unsuccessful penalty shots.

As shown in Figure 3, across all seasons and both gender groups combined, goalkeeper saves accounted for the predominant share of unsuccessful penalty outcomes. In the male cohort, 1,869 goalkeeper saves were recorded against 498 post and missed attempts across all seasons combined, yielding a goalkeeper-attributable failure rate of 78.9%. Among female penalties, 1,538 saves were recorded compared to 631 non-save failures, corresponding to a goalkeeper-attributable rate of 70.9%. Male goalkeepers consistently recorded higher save percentages than their female counterparts across all six seasons examined. In the 2017–18 season, male goalkeepers recorded a save percentage of 79.1% compared to 71.5% for female goalkeepers, establishing an initial gender differential of 7.6 percentage points. A notable convergence occurred in the 2023–24 season, where save percentages approached near-parity for the first time across the observation period: female goalkeepers recorded 70.3% and male goalkeepers 70.8%, a differential of only 0.5 percentage points. Female goalkeepers demonstrated greater seasonal variability over the same period, with save percentages ranging from 66.8% to 73.4%, while reaching their peak performance in 2021–22 (73.4%).

## Discussion

Performance in elite sport is shaped not only by technical execution but also by athletes' capacity to regulate cognitive, emotional, and physiological states under situational pressure. Using a multi-season dataset from elite handball, the present study examined how 7-m penalty performance is shaped by temporal factors, match context, and competitive pressure.

The temporal distribution of penalty occurrences aligns with previous research documenting increased penalty rates during specific match phases. Notably, the literature reports a penalty average of 2.5 per game during the 0–50 min period, escalating to 3.7 per game in the 50–60 min timeframe (Oikonomou et al., 2021), with further intensification in the final 10 min (Gómez et al., 2013). Analysis of closely contested matches in the Greek Men's Handball Championship corroborates this pattern, showing that 7-m penalty frequency increased from 2.5 in the 0–50-min period to 3.7 in the final 10 min of play. Previous research has attributed the increase in penalties during latter match stages to factors including player fatigue, mounting psychological pressure related to score differentials, and the urgency imposed by time constraints (Gómez et al., 2013; Oikonomou et al., 2021). The escalation of penalties in the final minutes likely reflects the compounding effects of physical exhaustion and the psychological stress associated with protecting or overcoming score deficits, leading defending teams to commit more infractions as they struggle to maintain defensive integrity under increasing pressure.

Also, the results of the current study bring out an average penalty success rate of 76.7% in the final 5 min, 76.0% in the last 10 min, and 76.4% across the entire match, aligning closely with previously documented conversion rates. Meletakos et al. (2011) reported stable success rates across major championships: 72.0% in 2005, 73.0% in 2007, and 71.2% in 2009, while more recent research indicates that success rates for 7-m penalties generally range between 68% and 84%, depending on game circumstances such as player experience and goalkeeper skills (Meletakos et al., 2024). The consistency observed in the present study falls within the upper range of these established benchmarks, suggesting that the analyzed leagues feature high-quality penalty execution. From a psychological perspective, the observed stability of 7-m penalty success rates across temporal windows and pressure conditions suggests effective regulation of cognitive and emotional states among elite players. While some theoretical models predict performance deterioration under stress, the present descriptive findings show that elite handball players maintained consistent execution during high-stakes moments. This pattern is consistent with contemporary views on neural efficiency and expert performance, whereby skilled athletes may demonstrate reduced performance variability and more economical cognitive processing under pressure (Cooke et al., 2014). The observed stability could reflect adaptive stress regulation in expert sport performance (Laborde et al., 2014), though alternative explanations, such as task familiarity, extensive training, or situational factors, cannot be ruled out without further investigation. Importantly, these findings may also reflect adaptive mechanisms characteristic of challenge situations, which share similar cognitive processing pathways with pressure conditions (Wang et al., 2021). Challenge situations, defined as demanding task conditions requiring continuous adjustment and controlled processing, activate what the multi-action plan model describes as an optimal-controlled state (Lu et al., 2025; Wang et al., 2019). In such states, expert performers demonstrate economical cognitive processing through a temporally organized neural sequence: initial engagement of motor control and selective attention (lower alpha power at posterior sites) to essential task components. This temporal switch between motor control and attentional processes reflects adaptive allocation of cognitive resources specific to challenging performance demands (Li et al., 2025; Lu et al., 2025; Wang et al., 2019).

Furthermore, performance in high-stakes penalty situations is shaped by multiple interacting constraints, including physiological factors such as VO2max thresholds and blood lactate concentration (Alkhawaldeh, 2022), tactical elements like deceptive shooting techniques (Melnyk et al., 2016), biomechanical precision required for effective shot execution (Van den Tillaar and Ettema, 2009), the thrower's ability to generate optimal ball velocity and accuracy (Demiray et al., 2025; García et al., 2017), shot type and target location selection, and contextual influences such as goalkeeper effectiveness under home vs. away conditions (Hantǎu and Hantǎu, 2014). The present findings can be interpreted in light of these multifactorial determinants.

In clutch moments of matches, penalty shot performance at the elite handball level is characterized by broad stability under pressure, with conversion rates in the final minutes of matches remaining largely within the range of whole-match averages across most seasons and both gender groups. The absence of consistent and substantial performance decrements across the majority of seasons and both gender groups suggests that elite handball players may largely execute penalty shots as highly automated motor routines that are relatively resistant to situational pressure at this level of expertise. However, it is important to emphasize that the present study does not include psychological or physiological measurements; therefore, any interpretation of attentional or cognitive mechanisms must be treated as speculative. Future research incorporating measures of anxiety, attentional focus, or psychophysiological arousal during penalty execution would be necessary to examine these mechanisms directly. In contrast, some previous research suggests that psychological pressure significantly impacts penalty efficacy (Alsharji, 2014), yet aligns with evidence from Bühren and Gabriel (2023), who conducted an analysis of over 5,500 penalty throws from the Bundesliga season and found that performance often improves during decisive moments, suggesting that elite players effectively channel pressure to their advantage. Their findings suggest that, contrary to expectations of performance degradation under pressure, penalty shot success rates remain remarkably stable across different match phases, with a slight tendency to improve in the final moments of competition.

Further, the analysis of penalty shot performance during close matches, penalty success rates consistently exhibit notable stability across seasons, with observed fluctuations remaining within a relatively narrow range. Also, the analysis reveals comparable failure rates between matches characterized by a close score differential and the aggregate of all matches across seven consecutive seasons. These findings indicate that penalty shot execution remains remarkably consistent across match competitiveness, suggesting that the situational pressure of close games does not significantly impair penalty takers' technical and psychological capacity. Oikonomou et al. (2021) investigated throw effectiveness in close handball matches in the Greek League and found significant tactical shifts during critical match phases, including increased elevated suspension and penalty frequencies during the final 10 min. This pattern aligns with the present study's observation of elevated penalty frequencies during latter match stages, reinforcing the interpretation that defensive desperation and tactical fouling become more prevalent as teams attempt to protect narrow leads or prevent high-percentage scoring opportunities. However, the consistency of penalty conversion rates in the current analysis suggests that the increased frequency of 7-m penalties during critical periods does not translate into diminished execution quality. Despite defending teams committing more infractions under pressure, attacking players maintain their composure and technical proficiency when executing the resulting penalty shots. This stability in performance, coupled with evidence that overall throw effectiveness actually increases during the final minutes of close matches (Oikonomou et al., 2021), indicates that elite handball players possess the psychological resilience necessary to perform optimally in high-stakes situations.

While goalkeeper effectiveness has been identified as a significant predictor of match outcomes in elite handball, the present analysis demonstrates that approximately three out of every four unsuccessful 7-m shots across elite handball competitions result from goalkeeper intervention, with men's leagues exhibiting marginally higher save proportions than women's leagues. These findings highlight that goalkeeper intervention is the primary factor contributing to unsuccessful penalty shots, accounting for approximately three out of every four misses. Moreover, goalkeepers in men's leagues appear to have a marginally higher effectiveness in saving penalties compared to their counterparts in women's leagues. While seasonal fluctuations are evident, notably a marked drop in the most recent men's season, the overall trend supports the central role of goalkeepers in penalty shot outcomes. The remaining unsuccessful penalties were attributed to non-save errors, such as hitting the post or missing the target entirely. However, Krawczyk et al. (2024) observed that goalkeeper effectiveness during 7-m situations may not singularly dictate match outcomes. In that study, goalkeeper save efficiency emerged as a predictor of both goal differential and match victory, yet it was evaluated alongside other offensive and defensive indicators such as shot efficiency from 6 and 9 m, foul frequency, and fast-break execution. These multivariate findings suggest that while goalkeeper performance at the 7-m line contributes meaningfully to overall defensive efficacy, its singular impact on match success may be diluted within the broader tactical and technical landscape of the game (Krawczyk et al., 2024). In another study, Hantǎu and Hantǎu (2014) identified a goalkeeper effectiveness of 17.21% for 7-m shots in the Romanian National Men's League during the 2012–2013 season, highlighting a considerably lower contribution of goalkeepers to penalty outcomes in that context. Notably, elite goalkeepers typically maintain a save rate of approximately 30% during these shots (Rojas et al., 2012), corresponding closely to the approximately 24% failure rate observed in the current analysis, thereby reinforcing the inherent advantage afforded to shooters due to limited goalkeeper response time and optimal shooting distance. Although theoretical considerations suggest that even elite goalkeepers rarely exceed a 50% save rate on penalties due to the inherent offensive advantage in such situations, our multi-season dataset indicates that goalkeeper impact on penalty misses may be greater than previously assumed. This aligns with expertise frameworks suggesting that performance in handball is highly position-specific (Williams and Ford, 2008) and underscores the specialized perceptual-motor and anticipatory skills goalkeepers require to disrupt high-probability scoring opportunities such as penalty shots. Consequently, the aggregate impact of penalty save performance appears marginal relative to goalkeeper effectiveness in the more frequent and tactically variable situations encountered during regular play, where save success rates demonstrate greater variability and cumulative influence on goal differential.

### Limitations and future directions

Several limitations should be acknowledged. First, the analysis was based on aggregated, event-level data and did not account for individual shooter or goalkeeper characteristics, such as experience, role specialization, or psychological profile, which may influence performance under pressure. Second, the study relied on publicly available match statistics and therefore could not incorporate direct psychophysiological or neurocognitive measures to allow a deeper examination of underlying self-regulation mechanisms. Third, the dataset was restricted to elite Danish handball leagues, which may limit the generalizability of the findings to other competitive levels, leagues, or cultural contexts.

Future research should aim to integrate individual-level performance data with psychological, psychophysiological, and neurophysiological indicators (e.g., heart rate variability, EEG, or attentional measures) to better capture the mechanisms supporting performance stability under pressure. Longitudinal and cross-league comparisons may further clarify how expertise, training history, and competitive context interact to shape adaptive performance regulation in elite sport.

## Conclusions

This multi-season analysis provides novel insight into the temporal and situational dynamics of 7-m penalty performance in elite handball. Across seven competitive seasons and all genders, penalty success rates remained remarkably stable despite variations in match context, competitive pressure, and game timing. In practice, training programmes should therefore incorporate pressure simulation specifically replicating the conditions of the final 5 min of close matches, such as fatigued states, score-differential awareness, and consequence-laden execution environments. Scenario-based practice, where penalties are taken under simulated high-stakes conditions with outcome consequences for the training group, may help consolidate the performance stability.

## Data Availability

Publicly available datasets were analyzed in this study. This data can be found here: https://doi.org/10.34740/kaggle/dsv/15147496.

## References

[B1] Alkhawaldeh I. M. (2022). Impact of VO2max on mechanical variables, lactic acid concentration, and shooting accuracy of the 7-meter throw among handball players. SPORT TK–Revista Euroamericana de Ciencias del Deporte 20:20. doi: 10.6018/sportk.531631

[B2] Alsharji K. E. (2014). Perceptual Training Effects on Anticipation of Direct and Deceptive 7-Meter Throws in Team Handball (Doctoral dissertation). University of Minnesota, Minneapolis. doi: 10.1080/02640414.2015.1039463

[B3] Beilock S. L. Carr T. H. (2005). When high-powered people fail: working memory and “choking under pressure” in math. Psychol. Sci. 16, 101–105. doi: 10.1111/j.0956-7976.2005.00789.x 15686575

[B4] Bühren C. Gabriel M. (2023). Performing best when it matters the most: evidence from professional handball. J. Quant. Anal. Sports 19, 185–203. doi: 10.1515/jqas-2022-0070

[B5] Cooke A. Kavussanu M. Gallicchio G. Willoughby A. McIntyre D. Ring C. (2014). Preparation for action: psychophysiological activity preceding a motor skill as a function of expertise, performance outcome, and psychological pressure. Psychophysiology 51, 374–384. doi: 10.1111/psyp.12182 24611889 PMC4285917

[B6] Debanne T. Laffaye G. Trouilloud D. (2018). Motivational orientations and performance in penalty throws during elite male team handball games. Scand. J. Med. Sci. Sports 28, 1288–1294. doi: 10.1111/sms.12995 29047173

[B7] Demiray Z. Makaraci Y. Duysak H. (2025). Short-term delayed effects of kinesio taping on muscular activity and throwing velocity in female handball players: a randomized, placebo-controlled, single-blind, crossover study. Percept. Mot. Skills 131:00315125251357631. doi: 10.1177/00315125251357631 40590357

[B8] García J. A. Menayo R. Del Val P. (2017). Speed–accuracy trade-off in a 7-meter throw in handball with real constraints: goalkeeper and level of expertise. J. Phys. Educ. Sport 17, 1172–1176. doi: 10.7752/jpes.2017.02134

[B9] Gómez M. A. Lago C. Pollard R. (2013). “Situational variables,” in Routledge Handbook of Sports Performance Analysis, eds. T. McGarry, et al. (London: Routledge), 259–269.

[B10] Gross J. J. (2015). Emotion regulation: current status and future prospects. Psychol. Inq. 26, 1–26. doi: 10.1080/1047840X.2014.940781

[B11] Hantǎu C. Hantǎu C. (2014). Study concerning the effectiveness of handball goalkeeper at the 7-m throws. Marathon VI, 27–31.

[B12] Huesmann K. Schorer J. Büsch D. Witt J. Loffing F. (2023). Expert goalkeepers' and coaches' views on anticipation and cue utilisation facing backcourt throws in handball goalkeeping. Front. Sports Act. Living 5:1215696. doi: 10.3389/fspor.2023.1215696 37877118 PMC10591308

[B13] Krawczyk P. Gajewski J. Labiński J. Szczerba M. Smoliński M. Bodasiński S. . (2024). Predictors of goal difference and match outcome in Polish handball PGNiG Superleague before, during, and after the COVID-19 pandemic: a regression analysis. Int. J. Perform. Anal. Sport 1–16. doi: 10.1080/24748668.2024.2410598

[B14] Laborde S. Lautenbach F. Allen M. S. Herbert C. Achtzehn S. (2014). The role of trait emotional intelligence in emotion regulation and performance under pressure. Pers. Individ. Dif. 57, 43–47. doi: 10.1016/j.paid.2013.09.013

[B15] Laxdal A. Byrkjedal P. T. Ivarsson A. Sigurgeirsson O. Haugen T. (2024). Fact or fiction? Examining the veracity of common myths related to 7-meter throws in handball. J. Hum. Kinet. doi: 10.31234/osf.io/q9fb6 PMC13215230 42211803

[B16] Li D. Elbanna H. Lin F.-Y. Lu C.-J. Chen L.-J. Lu G. . (2025). Neuromotor mechanisms of successful football penalty kicking: an EEG pilot study. Front. Psychol. 16:1452443. doi: 10.3389/fpsyg.2025.1452443 40458627 PMC12127302

[B17] Lu G. Hagan J. E. Jr. Cheng M.-Y. Chen D.-T. Lu C.-J. Lin F.-Y. . (2025). Amateurs exhibit greater psychomotor efficiency than novices: evidence from EEG during a visuomotor task. Front. Psychol. 16:1436549. doi: 10.3389/fpsyg.2025.1436549 40771319 PMC12327254

[B18] Makaraci M. Makaraci Y. Zorba E. Lautenbach F. (2024). Effects of ten biofeedback sessions on athletes' physiological, psychological, and cognitive functioning: a randomized controlled trial with international tennis players. Percept. Mot. Skills 131, 1664–1686. doi: 10.1177/00315125241274834 39149880

[B19] Meletakos P. Manasis V. Noutsos K. Bayios I. (2024). Performance differences and determinants of success in world men's handball championships. Motriz: Revista de Educação Física 30:e10240068. doi: 10.5016/s1980-6574e10240068

[B20] Meletakos P. Vagenas G. Bayios I. (2011). A multivariate assessment of offensive performance indicators in men's handball: trends and differences in the World Championships. Int. J. Perform. Anal. Sport 11, 284–294. doi: 10.1080/24748668.2011.11868548

[B21] Melnyk V. Pasichnyk V. Levkiv V. Kovtsun V. (2016). Tactical attacking actions of competitive handball players with different qualifications. J. Phys. Educ. Sport 16, 77–83. doi: 10.7752/jpes.2016.01013

[B22] Oikonomou C. Yannakos A. Acsinte A. (2021). The throw effectiveness in the last minutes of handball close games and the factors that influence it. Gymnasium 22, 121–130. doi: 10.29081/gsjesh.2021.22.2.09

[B23] Oliveira T. Gómez M.-A. Sampaio J. (2012). Effects of game location, period, and quality of opposition in elite handball performances. Percept. Mot. Skills 114, 783–794. doi: 10.2466/30.06.PMS.114.3.783-794 22913020

[B24] Rojas F. J. Gutiérrez-Dávila M. Ortega M. Campos J. R. Párraga J. (2012). Biomechanical analysis of anticipation of elite and inexperienced goalkeepers to distance shots in handball. J. Hum. Kinet. 34, 41–48. doi: 10.2478/v10078-012-0062-0 23487516 PMC3590836

[B25] Srhoj V. Rogulj N. Katić R. (2001). Influence of the attack end conduction on match result in handball. Coll. Antropol. 25, 611–617. 11811292

[B26] Van den Tillaar R. Ettema G. (2009). Is there a proximal-to-distal sequence in overarm throwing in team handball? J. Sports Sci. 27, 949–955. doi: 10.1080/02640410902960502 19629844

[B27] Vuleta D. Milanović D. Sertić H. (2003). Relations among variables of shooting for a goal and outcomes of the 2000 Men's European Handball Championship matches. Kinesiology 35, 168–183.

[B28] Wagner H. Pfusterschmied J. von Duvillard S. P. Müller E. (2011). Performance and kinematics of various throwing techniques in team handball. J. Sports Sci. Med. 10, 73–80. 24149298 PMC3737895

[B29] Wang K.-P. Cheng M.-Y. Chen T.-T. Chang Y.-K. Huang C.-J. Feng J. . (2019). Experts' successful psychomotor performance was characterized by effective switch of motor and attentional control. Psychol. Sport Exerc. 43, 374–379. doi: 10.1016/j.psychsport.2019.04.006

[B30] Wang K.-P. Frank C. Tsai Y. Lin K.-H. Chen T.-T. Cheng M.-Y. . (2021). Superior performance in skilled golfers characterized by dynamic neuromotor processes related to attentional focus. Front. Psychol. 12:633228. doi: 10.3389/fpsyg.2021.633228 33664700 PMC7921727

[B31] Williams A. M. Ford P. R. (2008). Expertise and expert performance in sport. Int. Rev. Sport Exerc. Psychol. 1, 4–18. doi: 10.1080/17509840701836867

